# Bio-Inspired Sutures: Simulating the Role of Suture Placement in the Mechanical Response of Interlocking Structures

**DOI:** 10.3390/biomimetics8070515

**Published:** 2023-10-31

**Authors:** Melissa M. Gibbons, Diana A. Chen

**Affiliations:** 1 Department of Mechanical Engineering, University of San Diego, San Diego, CA 92110, USA; 2 Department of Integrated Engineering, University of San Diego, San Diego, CA 92110, USA; dianachen@sandiego.edu

**Keywords:** bio-inspired, suture, mechanical properties, finite element model

## Abstract

The hardest anatomical components of many animals are connected at thin seams known as sutures, which allow for growth and compliance required for respiration and movement and serve as a defense mechanism by absorbing energy during impacts. We take a bio-inspired approach and parameterize suture geometries to utilize geometric connections, rather than new engineering materials, to absorb high-impact loads. This study builds upon our work that investigated the effects of the dovetail suture contact angle, tangent length, and tab radius on the stiffness and toughness of an archway structure using finite element analysis. We explore how increasing the archway segmentation affects the mechanical response of the overall structure and investigate the effects of displacement when induced between sutures. First, when keeping displacement along a suture but increasing the number of archway pieces from two to four, we observed that stiffness and toughness were reduced substantially, although the overall trends stayed the same. Second, when the displacement was induced along an archway edge rather than upon a suture (in a three-piece archway), we observed that archway stiffness and toughness were much less sensitive to the changes in the suture parameters, but unlike the archway indented along the suture line, they tended to lose stiffness and toughness as the tangent length increased. This study is a step forward in the development of bio-inspired impact-resistant helmets.

## 1. Introduction

**Figure 1:**
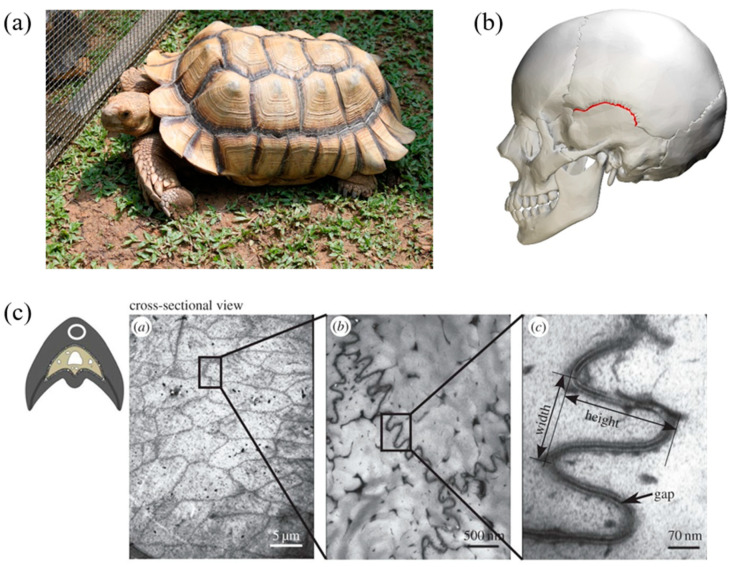
Examples of (**a**) a turtle carapace (Tortoise By The Net © Mark Yang, CC BY 2.0) and (**b**) a human skull (Squamosal suture—skull—lateral view © Was a bee, CC BY-SA 2.1 JP) featuring sutures between hard anatomical components. (**c**) Transmission electron microscope images of the cross-section of a red-bellied woodpecker beak showing the suture lines at (*a*) 5 μm, (*b*) 500 nm, and (*c*) 70 nm scales (adapted from [6], reprinted with permission).

Bio-inspired design draws upon strategies fine-tuned by Nature over 3.8 billion years and applies these lessons learned towards human-made artifacts to mimic the function of biological organisms [1,2,3]. In engineering structures, some of the mechanical parameters of interest include the stiffness and toughness of materials to uphold structural integrity in the face of expected and unexpected forces. Stiffness characterizes a material’s ability to resist deformation under an applied load, indicating its capacity to maintain its shape and structure under external pressure. Toughness denotes a material’s ability to absorb energy and deform plastically before failing, signifying its resilience and ability to endure sudden impacts or dynamic forces (i.e., damage tolerance) [4]. This study examines the role of sutures—found in a variety of species including in human skulls, woodpecker beaks, and turtle carapaces, among others—that consist of a soft interfacial material and whose function is to connect some of the hardest anatomical components in animals at thin seams (see Figure 1) [5,6,7]. The sutures not only allow for normal growth and the compliance required for respiration and movement but can also serve as a defense mechanism by absorbing energy during impact, preventing structural failures that would result in significant injuries to the animal [8,9].

One potential application of these sutures is to apply this principle to the development and optimization of protective helmets for dynamic impacts experienced by humans (e.g., sports and cycling). While helmet design is well-studied in mechanical engineering [10,11], this study explores if the addition of sutures and the modularization of the shield-like structure into components can provide additional protection through the energy-absorbing properties of the suture. The suture itself has the potential to be more than just connective tissue; its underlying geometry can play a large role in how high-impact loads are absorbed. For example, in a 1990 experimental study on goat cranial bone, Jaslow found that the geometric features of the suture morphology, including the degree of interdigitation and the length and width of the suture, affect the strength and impact energy absorption of the suture material [12]. The advantage of studying the geometry of the suture lies in utilizing the structural properties of the connection, rather than (or in addition to) relying on new engineering materials, to absorb high-impact loads. A similar strategy is being used to develop advanced ceramics based on bio-inspired architectures to improve and tune the mechanical response in multi-impact conditions [13].

For the purposes of this paper, we briefly summarize the literature upon which this suture exploration is founded. Researchers have studied the effect of both trapezoidal and round suture shapes on the mechanical response of flat pieces of material to in-plane loading. An analytical study examined trapezoidal suture geometries under tension perpendicular to the suture axis, under tension parallel to the suture axis, and under in-plane shear [14]. An experimental study examined trapezoidal suture geometries under tensile loading normal to the suture axis using 3D-printed test pieces [15]; the inclusion of sutures resulted in significant variation in stiffness, strength, and toughness, offering a chance to adjust the geometric characteristics for a targeted mechanical reaction. Malik et al. (2017) analytically examined round sutures subjected to tension perpendicular to the suture axis [16]. They adjusted the suture tab size by modifying the tab radius and managed interlocking via the contact angle. Maximum stiffness and strength were observed when the contact surface was frictionless, as increased friction heightened the risk of tab fracture. In a subsequent investigation, they increased the contact area by incorporating a dovetail-like component into the suture, introducing a straight-line segment between the protruding and recessed tabs [17]. This addition significantly boosted the structure’s toughness, underscoring the mechanical advantages of increasing the contact area. 

Malik et al. tested single sutures under normal in-plane tension [16,17]; our work builds upon theirs to study multiple sutures within a larger archway structure under out-of-plane displacement. We use the same geometric parameters to define the dovetail suture: (1) suture tab radius, which determines overall tab size, (2) contact angle, which determines the degree of interlocking, and (3) tangent length, which is the length of the straight-line segment introduced between the protruding and recessing tabs. 

In our previous finite element analysis (FEA) study [18], we simulated an out-of-plane bending load on an archway structure created from two symmetric 90° pieces that were joined together at the center by the interlocking dovetail suture. The two mechanical properties we focus on pertaining to helmet design are structural toughness—which is critical for absorbing impact energy to prevent loads from being transferred to the human head—and structural stiffness—which is important for resisting deformation and preventing the helmet itself from buckling and injuring the user [19]. It is important to note our emphasis on “structural”; we study how the change in just suture geometry (placement relative to the indenter, number of sutures along the archway, and suture tab parameters) affects the toughness and stiffness of the overall model. Our previous study sought to identify the optimal combination of suture parameters for maximizing toughness (quantified by the total strain energy in the archway) while not significantly sacrificing stiffness (quantified by the final contact force between the indenter and the archway); we found that, generally, the suture parameters that increased the degree of interlocking and contact surface area maximized the archway stiffness and toughness. Since the basic archway in the previous study consisted of two symmetrical pieces, the indentation occurred at the suture line. In this study, we increased the number of archway pieces, which affects where the vertical displacement is induced along the structure. Our goal is to seek which suture features (suture geometry but also indenter placement along either a suture or face) maximize the stiffness and toughness of the structure as a whole and to determine if there are designs that should be excluded based on the resulting mechanical response.

## 2. Methods

### 2.1. Archway and Suture Geometries

**Figure 2:**
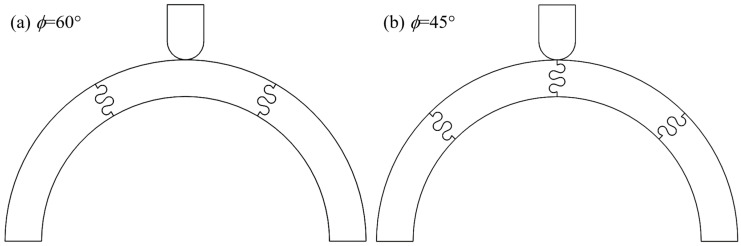
Archway structures were created with a varying number of component pieces, characterized by the revolved angle of each piece, denoted by *ϕ*. (**a**) Three archway pieces are joined when *ϕ* = 60° and (**b**) four archway pieces are joined when *ϕ* = 45°. The suture parameters shown in both archways are *θ* = 20°, *r* = 3 mm, and *L* = 3 mm.

To expand our work towards its intended application as an impact-resistant helmet, we modified the archway geometry in two distinct ways: first, we explored how increasing the number of segments along the archway affected the mechanical response of the overall structure (i.e., structural stiffness and toughness); second, we modified the archway structure to induce displacement between (rather than along) suture lines, as impact location is not fixed in reality. As shown in Figure 2, this parameter is characterized by the revolved angle of each piece of the archway, denoted by *ϕ* in this work. Note that when an odd number of suture lines are incorporated into the archway, the pieces are mirrored and repeated (in the case of *ϕ* = 45°), meaning the final structure is not strictly symmetrical. In the case of *ϕ* = 60°, the even number of suture lines allowed for the middle piece, and therefore the archway as a whole, to be symmetrical.

**Figure 3:**
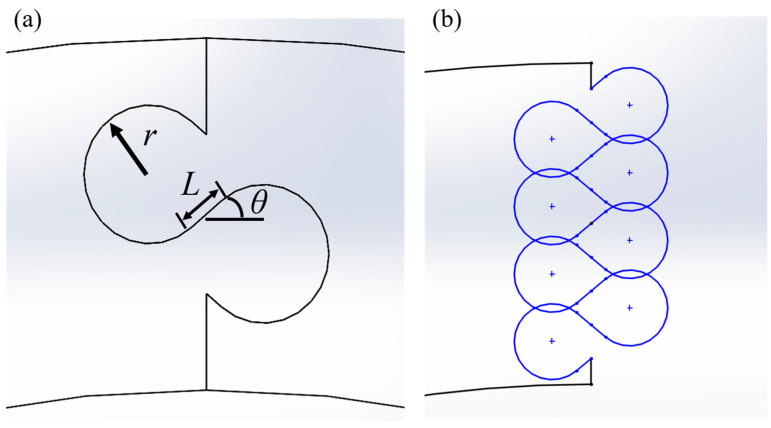
(**a**) Dovetail suture parameters that were varied: suture tab radius, *r*, tangent length, *L*, and contact angle, *θ*. Reprinted from [18]. (**b**) Example of a suture parameter combination (*θ* = 40°, *r* = 3 mm, and *L* = 3 mm) shown in draft format that is physically inadmissible due to the overlaps between suture tabs.

In the first study, which focused only on *ϕ* = 90° archways, we explored the entire range of physically admissible suture parameter values (Figure 3a). The contact angle, *θ*, varied from 0° to 40°, the suture tab radius, *r*, varied from 1 mm to 5 mm, and the tangent length, *L*, varied from 0 mm to 20 mm. Not all parameter combinations were studied as some produced physically impossible geometries due to suture tab overlaps (e.g., Figure 3b). Overlaps occur in the sutures with larger contact angles when the tangent length is increased; the effect is most pronounced when the suture tab radius is small. 

In the *ϕ* = 90° study, we found that several suture parameter values produced a suboptimal archway mechanical response (i.e., the resulting archways were less stiff and tough than when other suture geometries were used). A lack of a contact angle (*θ* = 0°), large suture tab radii (*r* > 3 mm), and large tangent lengths (*L* > 10 mm) produced suboptimal mechanical responses when the archway was indented along a suture line. We hypothesized that the response trends would be similar for the *ϕ* = 45°cases because loading was again applied along a suture edge. The preliminary findings indicated that the hypothesis was correct, so we excluded the *θ* = 0°, *r* > 3 mm, and *L* > 10 mm suture parameter values in this study, and we sampled just a few tangent length values within each remaining contact angle/suture tab radius combination. 

For the *ϕ* = 60°cases, we decided to explore the entire range of physically admissible suture parameter values due to the different loading condition. In cases where admissible geometries included a range of tangent lengths up to 20 mm, we simulated the tangent length in increments of 2 mm (i.e., 0 mm, 2 mm, … 18 mm, and 20 mm) to adequately sample the full range.

### 2.2. FEA Setup

**Figure 4:**
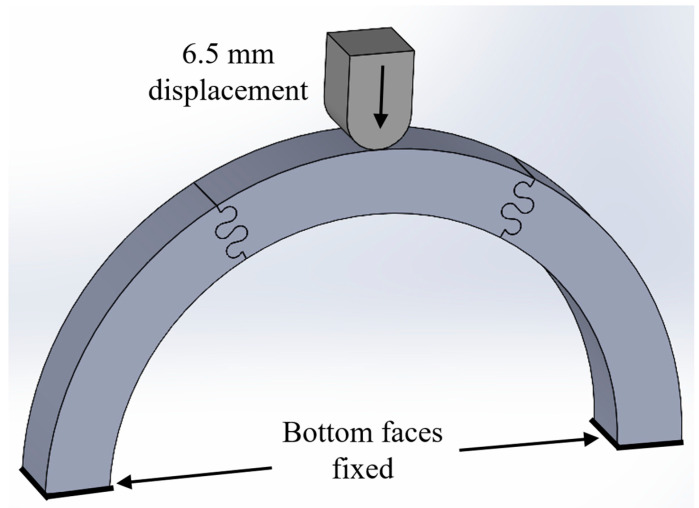
Representative model assembly for a *ϕ* = 60° archway. The indenter was vertically displaced 6.5 mm and the two bottom faces of the archway were fixed.

We created and solved all models in ANSYS Mechanical (V2022 R2; ANSYS, Inc., Canonsburg, PA, USA). The archways were created with an inner radius of 100 mm and a cross-sectional area of 25.4 mm × 25.4 mm to mimic the average adult head size and helmet liner thickness, respectively [20]. The archway pieces were modeled using polylactic acid (PLA), as pieces will later be 3D printed for future studies focused on dynamic impact loads. PLA has a modulus of elasticity, *E* = 2.94 GPa, and Poisson’s ratio, *ν* = 0.33. The indenter was modeled as stainless steel with a modulus of elasticity, *E* = 200 GPa, and Poisson’s ratio, *ν* = 0.3. Both materials were considered linearly elastic in our analysis, as the primary focus of this study was the structure’s response prior to material yield and failure. The two bottom faces of the archway assembly were constrained by a fixed boundary condition and a frictionless contact setting was applied between the archway segments. Malik et al. found that the presence of friction increased contact stresses in the suture tabs, which led to tab fracture [16,17]; frictionless contact is therefore ideal for the intended helmet applications. A quasi-static displacement-controlled simulation was performed in which the indenter was displaced a total of 6.5 mm to induce substantial deformation in the archway pieces and visible suture tab pull-out in many cases. The model setup for a representative archway is shown in Figure 4. Total strain energy and final contact force are output to produce comparable data to the first study.

A mesh convergence study was performed to determine appropriate settings on an archway with suture parameter values near the median of all values studied (*ϕ* = 60°, *θ* = 10°, *r* = 3 mm, and *L* = 6 mm). Automatic meshing in ANSYS Mechanical was used to create solid meshes with a mixture of quadratic hexahedral and tetrahedral elements, with a global element size of 3 mm to ensure that all features could be discretized without losing geometric detail. This initial mesh will be termed the coarse mesh. Contact sizing was applied at the suture contact points with element sizes of 1 mm (medium mesh) or 0.5 mm (fine mesh) to add more mesh detail in the areas with substantial curvature. As shown in Table 1, all three meshes produced converged global result outputs of interest. However, non-physical tab overlap occurred when the coarse and medium mesh settings were used. The fine mesh setting was necessary to eliminate this non-physical tab overlap; these mesh parameters were used for all simulations in this study.

**Figure 5:**
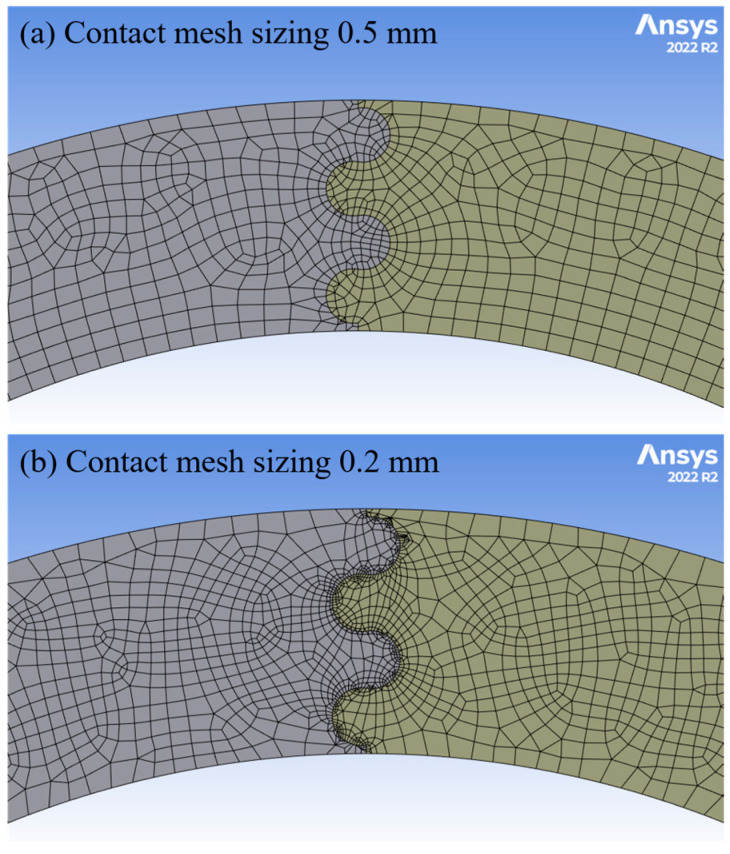
Examples of (**a**) the fine mesh produced with the contact mesh sizing of 0.5 mm used in most cases and (**b**) a finer mesh that was produced by decreasing the element size at the contacting surfaces to 0.2 mm.

The *ϕ* = 45° archway structure had two unconstrained segments, and in conjunction with the frictionless contact setting defined between individual segments, some simulations initially did not converge because the unconstrained pieces experienced out-of-plane rigid body motion. In these cases, it was necessary to add additional frictionless boundary conditions to the flat faces of the two unconstrained archway pieces to eliminate the out-of-plane motion of the part; this modification allowed the simulations to converge. We believe the simulations that converged without the additional boundary condition would not have turned out any different if constrained, since displacement was only induced in the vertical plane. The additional boundary condition was only necessary in a few cases where random numerical rounding errors led to the rigid-body motion. Despite also having an unconstrained segment, none of the *ϕ* = 60° archway simulations exhibited this behavior. As in the previous study, if the fine contact mesh settings described above resulted in substantial suture tab overlap as the indenter displacement increased or outright failure due to lack of convergence, the contact sizing was decreased further to 0.2 mm to produce a finer mesh at the contacting surfaces (see Figure 5), while the global mesh size of 3 mm was retained. These two modifications resolved most numerical issues. We excluded any cases that failed or exhibited numerical issues after these adjustments (which only applied to one case from the *ϕ* = 45° set).

## 3. Results and Discussion

### 3.1. Base Cases: Verifying the Role of Sutures

First, as a sanity check, we simulated ‘base cases’ in each *ϕ* category to confirm that sutures do play a significant role in increasing stiffness and toughness compared to no sutures. These base cases featured flat faces at the contact areas between archway pieces, with frictionless contact settings for comparison purposes. The results from the suture simulations are discussed relative to these base cases below.

**Figure 6:**
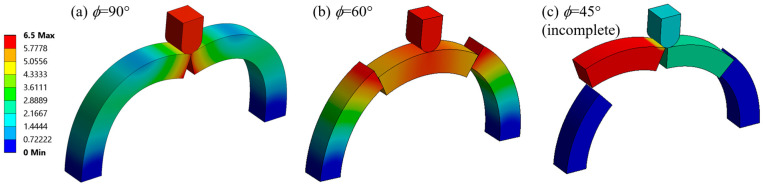
Deformed archway structure for the three base cases. (**a**) *ϕ* = 90° (reprinted from [18]) and (**b**) *ϕ* = 60° are shown at 6.5 mm total indenter displacement, and (**c**) *ϕ* = 45° is shown at an indenter displacement of 2 mm, when the simulation failed.

The final deformations of the base cases (Figure 6) visually support the conclusion from previous studies that the sutures play an important role in holding the structure together and transferring loads between archway pieces (i.e., increasing the stiffness and toughness of the structure). In particular, in the *ϕ* = 60° and *ϕ* = 45° base cases, the unconstrained pieces can be seen sliding out of place in Figure 6, which does not occur when sutures are present. Only numerical results from two of the three base cases (*ϕ* = 90° and *ϕ* = 60°) are shown and discussed below; results are not shown for the structure with four (*ϕ* = 45°) archway pieces with flat surfaces because the simulation was unable to converge even after the additional frictionless boundary conditions were applied on all four out-of-plane flat faces of the unconstrained pieces. Because the center piece was clearly sliding between the two support pieces (Figure 6b), the *ϕ* = 60° base case would have failed if the indenter displacement was increased much further. 

### 3.2. Comparison of ϕ Values

**Figure 7:**
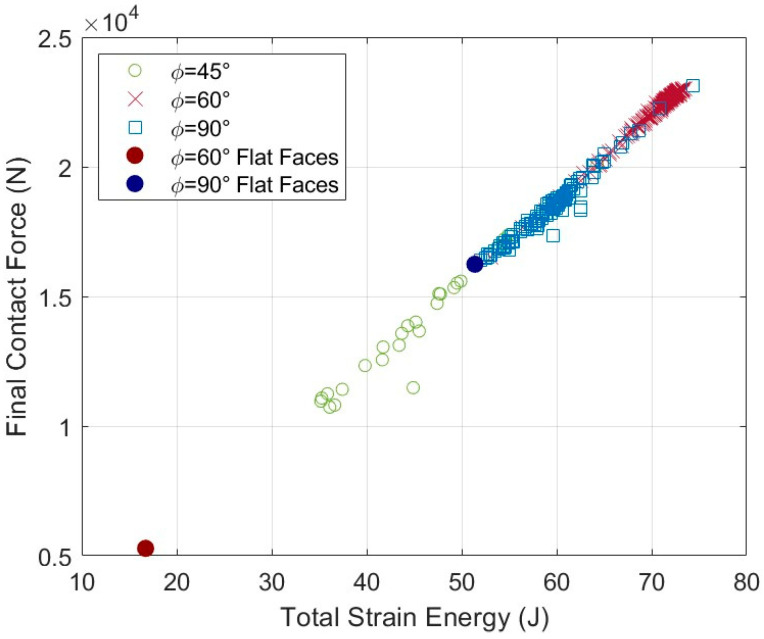
Final contact force (N) versus total strain energy (J) for all cases.

**Figure 8:**
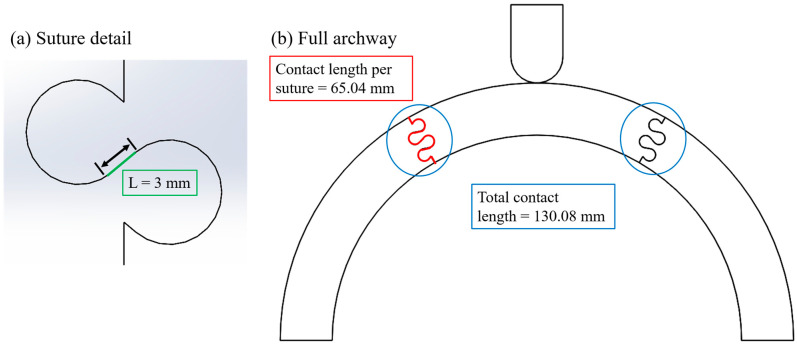
Illustrations of the different length parameters used for plotting purposes. (**a**) Suture detail showing the tangent length, *L*, in this case 3 mm (*L* was varied from 0–20 mm in the study). (**b**) Full archway showing the contact length per suture in red, in this case 65.04 mm, and the total contact length, which is a multiple of the contact length per suture. In this representative *ϕ* = 60° archway, the total contact length is two times the contact length per suture or 130.08 mm.

The final contact force from each successful case is plotted against the total strain energy in the archway in Figure 7, where the results are grouped by *ϕ*. Out of 163 total cases from the original *ϕ* = 90° study, 114 of them are presented in this study to aid comparison between the *ϕ* datasets (i.e., half-millimeter tangent length increments were removed since they were out of the scope of this study). There were 26 successful cases for *ϕ* = 45° and 189 successful cases for *ϕ* = 60°. Numerical results from the base cases, which are included in Figure 7, indicate that the archways without sutures are indeed less stiff and less tough than archways with sutures (i.e., the base cases appear at the lower end of their *ϕ* range). The *ϕ* = 60° base case was by far the least stiff and least tough of all cases that were successfully simulated in the study. As in the last study, it is interesting to note that stiffness and toughness continue to appear linearly related in the new *ϕ* cases. Moving forward, only final contact force results are presented with the understanding that the total strain energy results follow the same trends. The results of our simulations are illustrated using two dependent variables of interest (final contact force and total strain energy) as a function of geometric parameter inputs. The independent variables we focus on in the graphs below are all variations of length, all illustrated in Figure 8: the tangent length, *L*, of an individual suture tab; the contact length per suture, which is found from the total path length of the suture from the exterior surface to the interior surface of the archway; and the total contact length, which is a multiple of the contact length per suture that accounts for the total number of sutures in the archway structure (one for *ϕ* = 90°, two for *ϕ* = 60°, and three for *ϕ* = 45°). (See [18] for a more detailed discussion on the relationship between these variables.) The results are not displayed as a function of suture tab radius or contact angle because the smaller amount of discrete radius and angle values used in the study illustrates the same data in more compacted views (i.e., trends are harder to observe on the plots). 

**Figure 9:**
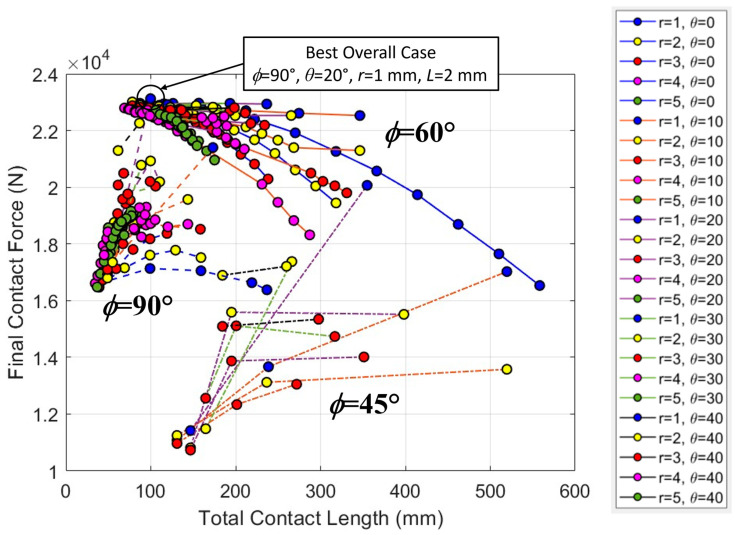
The final contact force (N) versus total contact length (mm) for all cases illustrates clear groupings based on *ϕ* value. The legend indicates the line and marker color used for each contact angle and suture tab radius combination, which were kept consistent across all three *ϕ* datasets. Solid lines are used for *ϕ* = 60° cases, dashed lines are used for *ϕ* = 90° cases, and dash–dotted lines are used for *ϕ* = 45° cases.

Figure 9 shows the final contact force from all simulations as a function of the total contact length for each archway structure. The groupings by *ϕ* are evident in the figure; the spread between them is emphasized by plotting the results as a function of total contact length, which for a given suture geometry increases by a factor of two or three from *ϕ* = 90°, when *ϕ* = 60° or *ϕ* = 45°, respectively. While the suture tangent length, *L*, is not explicitly shown in the figure, the total contact length is roughly correlated to the tangent length, with increases in *L* producing increases in the total contact length. On average, the *ϕ* = 60° archways were the stiffest, the *ϕ* = 90° archways were moderately stiff, and the *ϕ* = 45° archways were the least stiff. It should be noted that the overall stiffest geometry did come from the *ϕ* = 90° dataset (final contact force of 23,124 N). These broad results indicate that the structure performs better when it is not indented on the suture line. We discuss the three different groupings separately in more detail below.

### 3.3. Increasing Segmentation

**Figure 10:**
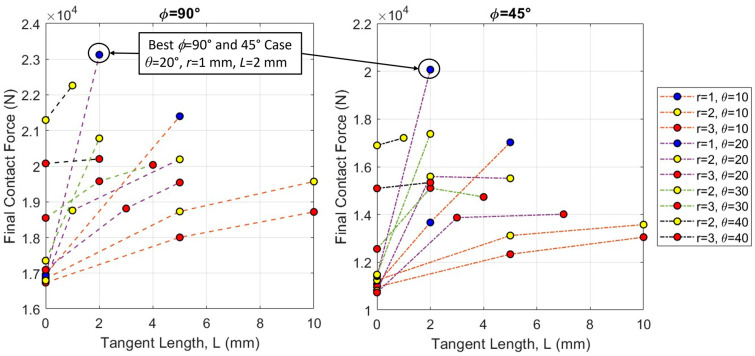
Comparison of *ϕ* = 90° and *ϕ* = 45° datasets illustrating very similar trends as a function of suture parameters (*r*, *L*, and *θ*). Note that the absolute final contact force values (N) are higher in *ϕ* = 90° than *ϕ* = 45°. Only a subset of the complete *ϕ* = 90° dataset (n = 25) is shown for comparison with the *ϕ* = 45° dataset (n = 26).

If we look closely at the two datasets where the archway is indented on a suture (Figure 10), we see that the *ϕ* = 45° dataset closely followed the trends illustrated in the *ϕ* = 90° dataset, but at lower final contact forces and total strain energy levels (not shown, as the trends are the same). Figure 10 shows the final contact force results for a subset of the *ϕ* = 90° dataset (n = 25) compared to the full *ϕ* = 45° dataset (n = 26). In general, the final contact force increased with increasing tangent length and contact angle and decreased with increasing suture tab radius, although plateaus and even slight drops in the force were observed in some cases as the tangent length increased. We found that the stiffest and toughest overall archway was from the *ϕ* = 90° dataset, with a suture tab geometry of *θ* = 20°, *r* = 1 mm, and *L* = 2 mm. This suture parameter combination also produced the best structural response in the *ϕ* = 45° dataset. In general, the *ϕ* = 45° archways produced the same trends in stiffness and toughness that we observed in the *ϕ* = 90° archways, except at substantially lower values. This suggests that increasing the segmentation of the archway does not improve the stiffness and toughness of the overall structure when it is indented along a suture line. 

### 3.4. Mid-Segment Indentation

**Figure 11:**
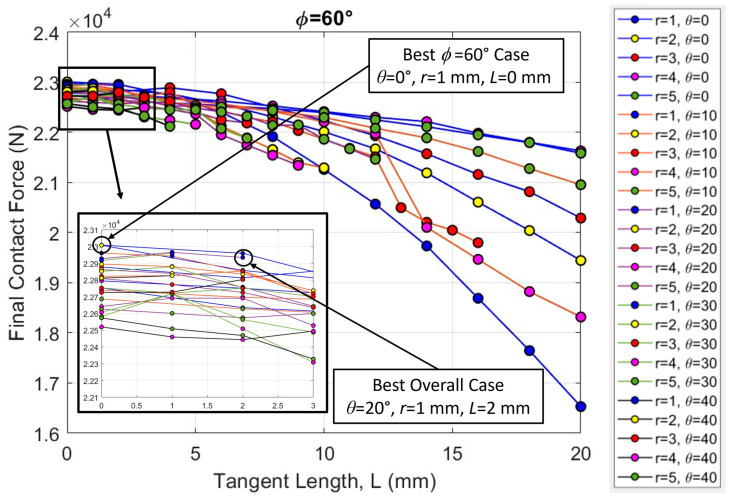
Final contact force (N) versus total contact length (mm) for all cases in the *ϕ* = 60° dataset. The inset shows the suture tangent length of 0–3 mm data points to visually separate the results.

If we look closely at the *ϕ* = 60° dataset where the archway is not indented on a suture (Figure 11), we see that there is a consistent downward trend in the final contact force with increasing tangent length. The force values are also quite compact, indicating that the overall archway stiffness is not as sensitive to the tab radius and contact angle as the *ϕ* = 45° and *ϕ* = 90° archways are. Both of these trends are substantially different from the trends observed in the datasets in which the archway was indented on a suture. The stiffest case in the *ϕ* = 60° dataset was found from a suture geometry of *θ* = 0°, *r* = 1 mm, and *L* = 0 mm, with a final contact force of 23,009 N, but this geometry actually resulted in one of the worst outcomes (i.e., the lowest final contact force) in the full *ϕ* = 90° dataset (16,604 N). Note that the final contact force from the suture geometry of *θ* = 20°, *r* = 1 mm, and *L* = 2 mm (the best overall case from Figure 10) in the *ϕ* = 60° dataset (22,935 N) was not substantially lower than the highest overall value (from the *ϕ* = 90° dataset) (23,124 N). The relative proximity of the best *ϕ* = 60° case to the best overall case is emphasized by the clumping of data observed in Figure 11 at values *L* < 5 mm; in general (and particularly at low tangent lengths), the results of the *ϕ* = 60° archways are not as sensitive to variations in suture parameters as the other *ϕ* datasets.

**Figure 12:**
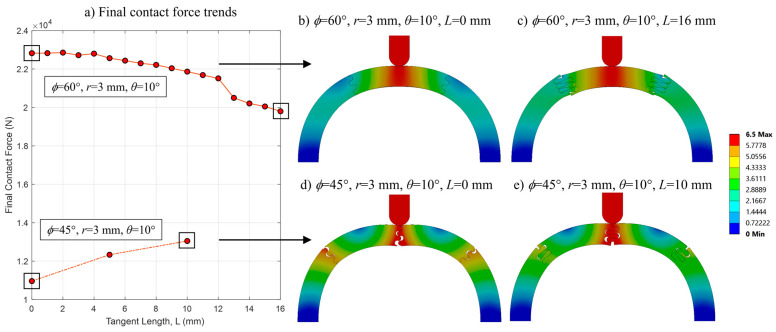
(**a**) Final contact force (N) versus tangent length (mm) for two representative curves from the *ϕ* = 45° and *ϕ* = 60° datasets; the corresponding deformed archway structures at 6.5 mm indenter displacement for the four boxed cases are shown in (**b**–**e**).

The final contact force generally increased with tangent length when indented on the suture line (*ϕ* = 45° and *ϕ* = 90°) and decreased with tangent length when indented between the suture lines (*ϕ* = 60°). Several representative final contact force curves from the *ϕ* = 45° and *ϕ* = 60° datasets were isolated to explain this result (Figure 12a). When there is no tangent length (*L* = 0 mm), there is visible separation between the individual pieces in the *ϕ* = 45° case (Figure 12d), and the ease with which the segments are able to separate results in a weak structure. When a tangent length is introduced (and elongated), the increased contact area at the sutures restores some of the load transference of the overall archway, which is illustrated by the reduction in deformation (i.e., more continuity) seen at the sutures (Figure 12e). In contrast, the archway pieces remain in contact in the *ϕ* = 60° case when there is no tangent length (Figure 12b); the load is evenly and effectively distributed to the two support pieces on either side (i.e., the archway is continuous). When a tangent length is included (Figure 12c), the elongated suture tabs allow for more localized relative displacement between the pieces (i.e., the segments act more independently), allowing the center piece to flatten as it is indented from above. This softening effect (the drop in final contact force) was amplified when the number of suture tab repeats increased from two to three when the tangent length was increased from 12 mm to 13 mm. (See [18] for more details about the effect of increasing suture repeats.)

The best overall suture parameter combination performed very well when *ϕ* = 60°. In absolute terms, the *ϕ* = 60° archways tended to be structurally stiffer and tougher than the *ϕ* = 45° and *ϕ* = 90° archways with the same suture tab geometry parameters, though this is likely attributed to the placement of the indenter on a face rather than directly upon a suture line. The stiffness and toughness of the *ϕ* = 60° archways were also much less sensitive to changes in the suture tab contact angle and radius. We conclude that future helmet/archway designs should focus on designing for the weaker case (*ϕ* = 90°) to be conservative for safety concerns. Then, if/when the impact falls upon a face rather than a suture (i.e., activating a situation similar to the *ϕ* = 60° configuration), the structure will be stiffer and tougher than minimally needed. The stiffness and toughness of the *ϕ* = 60° archways had a downward trend with increasing suture tangent length while *ϕ* = 45° and *ϕ* = 90° archways had an upward trend with increasing suture tangent length, suggesting that careful attention needs to be paid to optimizing the response when accounting for impacts on varying locations.

### 3.5. Reflections on the Role of Sutures in Localizing Strain Energy

The results indicated that increasing the segmentation of the archway (by decreasing the revolved angle, *ϕ*) did not improve the structural stiffness and toughness of the archway response as a whole. This result is not surprising when considering the integrity of the entire structure; the reduction in structural integrity with increased segmentation is exemplified by the deformation of the base cases shown in Figure 6 (even though the addition of sutures—especially, the optimal sutures as identified above—mitigates the instability). However, while the *ϕ* = 45° archways were not found to be inherently better than the *ϕ* = 90° archways because of the addition of more sutures, they were better at keeping the strain energy localized to the indented area. 

Consider a solid archway, an archway composed of two pieces (*ϕ* = 90°), and an archway composed of four pieces (*ϕ* = 45°). In the solid archway, any impact energy is transferred throughout the piece and into the ground to which it is fixed. In the *ϕ* = 90° archway, the two archway pieces distribute the load evenly and again transfer the energy into the ground to which it is fixed. In the *ϕ* = 45° archway, however, the two additional suture lines act as barriers that prevent a portion of the load from being transferred into the ground to which it is fixed. In essence, the two indented archway pieces are ‘sacrificed’—the nearby sutures isolate the prospective damage to the system to a local area and salvage the rest of the structure. 

To quantify this localization effect, we collected preliminary data on the amount of strain energy stored in the indented archway pieces. We hypothesize that if the percentage of the total strain energy stored in the indented archway pieces is greater than the percentage of their volume in the archway, then the sutures must play a role in localizing the damage. For example, the two indented pieces of a *ϕ* = 45° archway comprise 50% of the archway’s volume. The one indented piece of a *ϕ* = 60° archway comprises 33% of the archway’s volume. (The two indented pieces of a *ϕ* = 90° archway comprise 100% of the archway’s volume, so the percentage of the total strain energy stored in the indented archway pieces is always 100%). Preliminary data collected from 42 *ϕ* = 60° cases and 10 *ϕ* = 45° cases revealed that the indented archway pieces carried 33–54% more strain energy than their volume in the *ϕ* = 60° archways, and 3–38% more strain energy than their volume in the *ϕ* = 45° archways. For instance, the center piece of the *ϕ* = 60° archway with *θ* = 40°, *r* = 4 mm, and *L* = 1 mm carried 51% of the entire archway’s strain energy, although we would only expect it to absorb 33% of the strain energy if loads were to be evenly distributed. This archway absorbed the most relative strain energy in our preliminary dataset (0.51/0.33 = 1.54, or 54% more strain energy than expected). It is important to revisit the role of sutures in natural organisms to understand the significance of these results. Sutures found in biological systems allow for growth and movement, and they help to absorb energy during impact by preventing the energy from transferring drastically throughout the system. 

## 4. Conclusions

This study investigated the role of suture placement in the mechanical response of the archway under bending loading. The response of the structure based on where loads are applied is particularly relevant to helmet design, as the user has no way to predict where the head impact may occur. One might hypothesize that different suture parameter combinations would perform better under different segmentation configurations. However, in exploring the increasing segmentation of the archway along with the placement of the sutures, we identified a promising suture parameter combination of *θ* = 20°, *r* = 1 mm, and *L* = 2 mm that exhibited substantial toughness and stiffness in all three archway configurations that were studied. Thus, our future work will use the optimal suture parameter combination identified in this paper and shift our scope of study to other aspects of interest. 

One significant finding from this study was the similarity between the *ϕ* = 90° and *ϕ* = 45° datasets. This finding suggests that further segmentation of the archway would not provide new information, but would, rather, likely result in similar overall trends to those we have already identified, albeit at weaker values. For instance, we can expect *ϕ* = 30° (six segments) to follow the same trends as *ϕ* = 90° (two segments) and *ϕ* = 45° (four segments), which are all indented on a suture, and that *ϕ* = 36° (five segments) would follow the same trends as *ϕ* = 60° (three segments), which are both indented on a face, and so on. In conjunction with the promising suture parameter combination detailed above, the similarity between the datasets leads us to conclude that continued studies on further increasing segmentation would not add new knowledge about segmentation’s effect on the overall structural response.

In addition, we determined that increasing the archway segmentation results in a trade-off between the overall structural response and the localization of strain energy due to potential impact. While increasing the archway segmentation was found to reduce the archway stiffness and toughness, the preliminary results indicated that a larger fraction of the strain energy was absorbed by the impacted pieces when the archway segmentation was increased.

In future work, we will further explore the strain energy localization effect on archways with even greater segmentation in both indentation configurations. We hypothesize that while increasing segmentation will decrease structural stiffness and toughness, it will increase the strain energy localization effect throughout the structure. More segmentation suggests that smaller pieces of the archway can be sacrificed during an impact, and in turn, more of the archway can be salvaged by isolating the strain energy close to the area of impact and away from the edges of the archway (i.e., load transfer regions to adjacent bodies). We believe that the more segmentation there is, the better the structure will be at isolating damage due to the multiple sutures that the load needs to transfer through.

With this study, we conclude our work on investigating the role of suture parameters (*r*, *L*, and *θ*) on structural stiffness and toughness and identifying the optimal suture parameters based on indenter configuration. Our long-term goal is to create complex three-dimensional (3D) structures using modular building blocks that incorporate optimized suture geometries to aid in energy absorption. Future work quantifying the strain energy localization effect will allow us to determine the optimal size of the individual segments that will be used to create the helmet. In the context of the intended helmet application, the trade-off that exists between the reduced structural stiffness and toughness and the increased strain energy localization requires us to fully explore the parameter space to determine the segment size that maximizes both. This study and our future work contribute to our long-term goal of applying these suture geometries to helmet applications. 

## Figures and Tables

**Table 1:** Mesh convergence results for a representative archway (*ϕ* = 60°, *θ* = 10°, *r* = 3 mm, and *L* = 6 mm). The percent change is calculated as the difference between the coarse and medium mesh results and the medium and fine mesh results.

	Mesh Parameters	Nodes (Qty)	Elements (Qty)	CPU Time (s)	Final Contact Force (N)	% Change	Total Strain Energy (J)	% Change
Coarse	Global element size: 3 mm	58,527	12,195	397	22,371	N/A	70.674	N/A
Medium	Global element size: 3 mm Contact sizing: 1 mm	169,030	52,490	1141	22,400	0.13%	70.906	0.33%
Fine	Global element size: 3 mm Contact sizing: 0.5 mm	457,715	159,522	6940	22,437	0.17%	71.221	0.44%

## Data Availability

The data presented in this study are openly available in FigShare [21].
